# Intermittent Bi-Daily Sub-cutaneous Teriparatide Administration in Children With Hypoparathyroidism: A Single-Center Experience

**DOI:** 10.3389/fped.2021.764040

**Published:** 2021-11-08

**Authors:** Julie Bernardor, Sacha Flammier, Sara Cabet, Sandrine Lemoine, Roland Chapurlat, Arnaud Molin, Aurélia Bertholet-Thomas, Justine Bacchetta

**Affiliations:** ^1^Centre de Référence des Maladies Rares du Calcium et du Phosphore, Centre de Référence des Maladies Rénales Rares, Filières de Santé Maladies Rares OSCAR, ORKID et ERKNet, Service de Néphrologie Rhumatologie et Dermatologie Pédiatriques, Hôpital Femme Mère Enfant, Bron, France; ^2^INSERM UMR S1033 Research Unit, Lyon, France; ^3^Service de Néphrologie Pédiatrique, CHU de Nice, Hôpital Archet, Nice, France; ^4^Faculté de Médecine, Université Côte d'Azur, Nice, France; ^5^Département de Radiologie Pédiatrique et Fœtale, Hôpital Femme Mère Enfant, Lyon, France; ^6^Service d'Exploration Fonctionnelle Rénale, Centre de Référence des Maladies Rares du Calcium et du Phosphore, Centre de Référence des Maladies Rénales Rares, Filières de Santé Maladies Rares OSCAR et ORKID, Département de Néphrologie, Hôpital Edouard Herriot, Lyon, France; ^7^Faculté de Médecine Lyon Est, Université de Lyon, Lyon, France; ^8^Service de Rhumatologie, Hôpital Edouard Herriot, Lyon, France; ^9^Université de Normandie, UNICAEN, Unité de génétique, EA7450 BioTARGen, CHU de Caen Normandie, Caen, France

**Keywords:** children, hypoparathyroidism, nephrocalcinosis, phosphate, teriparatide

## Abstract

**Introduction:** The use of teriparatide has been reported in children with hypoparathyroidism as an investigational physiologic replacement therapy.

**Methods:** We aimed to retrospectively report our pediatric experience of bi-daily sub-cutaneous teriparatide. Results are presented as median (25th−75th quartile). As part of the routine follow-up of these patients with hypoparathyroidism, total calcium at H0 (i.e., just before injection) and H4 (i.e., 4 h after teriparatide injection) and other biomarker parameters were regularly assessed.

**Results:** At a median age of 10.7 (8.1–12.6) years, an estimated glomerular filtration rate (eGFR) of 110 (95–118) mL/min/1.73 m^2^, calcium levels of 1.87 (1.81–1.96) mmol/L and an age-standardized phosphate of 3.8 (2.5–4.9) SDS, teriparatide therapy was introduced in 10 patients at the dose of 1.1 (0.7–1.5) μg/kg/day (20 μg twice daily), with further adjustment depending on calcium levels. Six patients already displayed nephrocalcinosis. Severe side effects were reported in one child: two episodes of symptomatic hypocalcemia and one of iatrogenic hypercalcemia; one teenager displayed dysgueusia. Calcium levels at H0 did not significantly increase whilst calcium at H4 and phosphate levels significantly increased and decreased, respectively. After 12 months, eGFR, calcium and age-standardized phosphate levels were 108 (90–122) mL/min/1.73 m^2^, 2.36 (2.23–2.48) mmol/L, 0.5 (−0.1 to 1.5), and 68 (63–74) nmol/L, respectively, with a significant decrease in phosphate levels (*p* = 0.01). Urinary calcium and calcium/creatinine ratio remained stable; no nephrolithiasis was observed but two moderate nephrocalcinosis appeared.

**Conclusion:** Intermittent teriparatide therapy significantly improves calcium and phosphate control, without increasing calciuria. It appears to be safe and well-tolerated in children.

## Introduction

Pediatric hypoparathyroidism is an orphan disease with a prevalence in the United States and Europe ranging between 23 and 37 per 100,000 individuals (1). The association of hypocalcemia with an inappropriately low (or low-normal) PTH and hyperphosphatemia leads to the diagnosis (2). In children, etiologies of hypoparathyroidism are mostly genetic disorders which can be divided into three main categories: (1) disorders of parathyroid development, (2) disorders of parathyroid function, and (3) acquired parathyroid dysfunction (Table 1).

**Table 1:** Etiologies of pediatric hypoparathyroidism.

**Disorders of parathyroid development**	**Disorders of parathyroid function**	**Acquired parathyroid dysfunction**
- DiGeorge syndrome - Hypoparathyroidism Deafness Renal hypo-dysplasia (*GATA*3) - Autoimmune polyendocrinopathy-candidiasis-ectodermal dystrophy (APECED) syndrom (*AIRE*) - Other mutations: *GCMB, KCS1, TBCE*, or *FAM111A* genes	- Activating mutations in the Calcium Sensing Receptor (*CaSR or GNA11*)—Mutation in the *PTH* gene—Mitochondrial diseases (Kearns-Sayre syndrome)	- Post-surgery, radiotherapy—Anti-CaSR antibodies with or without APECED syndrome—Parathyroid infiltration: hemochromatosis or Wilson disease
		

The conventional management combines native vitamin D supplementation, active vitamin D analogs, and sometimes calcium supplementation. The aim of the management is to reach calcium levels within the lower normal range so as to avoid clinical signs and/or complications of hypocalcemia. It is crucial to avoid “over-treating” these patients by fully correcting calcium levels, the risk being to induce nephrolithiasis and/or nephrocalcinosis on the long-term because of hypercalciuria (2). Therefore, this management is a daily challenge for physicians mainly because of the control of hypocalcemia that can be difficult, and because of bad compliance. Furthermore, hyperphosphatemia increases morbi-mortality and should also be considered to avoid long-term consequences of hyperphosphatemia, and notably vascular calcifications and cardio-vascular comorbidities (3). The potential long-term cardiovascular consequences explain why phosphate binders are sometimes proposed to patients with hypoparathyroidism (2).

Ideally, the treatment of hypoparathyroidism replaces the absence or insufficiency of PTH. Such a treatment, namely recombinant human PTH, rhPTH (1–34) or teriparatide, has been first developed and approved in adults with osteoporotic fractures (4). It has also been used off-label as investigational physiologic replacement therapy in Europe and USA to treat adults with hypoparathyroidism who cannot be controlled by standard therapy (5). Synthetic and recombinant PTH 1–34 have been reported rather with daily or bi-daily subcutaneous administrations; moreover it was also reported using continuous subcutaneous infusion in adults first and then in children with hypoparathyroidism (6–16). In 2008, a randomized controlled study including 14 children with hypoparathyroidism has demonstrated that teriparatide is a safe and effective alternative to calcitriol therapy, being able to maintain normal serum calcium levels without hypercalciuria.

We aimed to retrospectively report our experience of bi-daily sub-cutaneous teriparatide in 10 children having received teriparatide as a second-line therapy for hypoparathyroidism. We hypothesized that twice-daily teriparatide therapy improves calcium and phosphate control.

## Patients and methods

### Patients

This is a retrospective single-center series of 10 children followed in our Reference Center for Rare Diseases of Calcium and Phosphate Metabolism in Lyon University Hospital for hypoparathyroidism. We included all children who started teriparatide therapy between April 2016 and June 2019 because of a failure of conventional therapy, with at least 1 year of follow-up.

### Teriparatide Introduction

As proposed by the French guidelines on hypoparathyroidism, teriparatide use was discussed as off-label second line therapy in children and teenagers in whom hypoparathyroidism was not well-controlled, either because of a problem of compliance (inducing recurrent symptomatic hypocalcemic episodes and/or severe uncontrolled hyperphosphatemia despite nutritional advice and phosphate binders) either because of recurrent symptomatic hypocalcemic episodes due to the severity of the underlying disease (and notably APECED syndromes). This management was always discussed by the two senior consultants of the reference center (ABT and JBa), with parents and children. The exact indication for teriparatide is displayed in Table 2. First, we introduce teriparatide in hospitalization, with a progressive withdrawal of conventional therapy during the first 24–48 h and controls of calcium levels just before the administration of teriparatide, and 4 h after. The first time point just before teriparatide administration helps us to assess the minimal dose required to be in the “safe zone” for hypocalcemia (our fasting target being between 1.8 and 2.0 mmol/L) in the absence of clinical symptoms of hypocalcemia. The second time point 4 h after teriparatide administration helps us to check that calcium levels are not too high, our target at this time point being between 2.0 and 2.2 mmol/L. Second, after the initial hospital stay, we follow total calcium and phosphate levels before and 4 h after administration and urinary calcium/creatinine on a spot every 2 weeks; when everything is controlled we only monitor calcium and phosphate levels before teriparatide administration. We usually see patients every 3 months with a complete biochemical evaluation, and we evaluate if possible 24 h-urinary calcium every 6 months, with a renal ultrasound at least yearly. Third, it is really important to maintain 25-D levels around 100 nmol/L so that teriparatide is fully efficient. In that setting, we supplement them regularly on the basis on 25-D levels, and the annual cumulative dose appears to be higher than in general pediatric populations. Fourth, it seems crucial that children have nutritional calcium intake within the target range for age (17). Last, it is of utmost importance to provide a specific therapeutic education to children and their caregivers (regular administration, signs of hypocalcemia) and on the fact that teriparatide should always be conserved between 2 and 8°C (risk of rapid inactivation). Children and their caregivers should always have active vitamin D analogs and calcium supplements with them in case of symptoms of hypocalcemia, even after injection, needing to change teriparatide cartridge: as such, an “emergency protocol” being explained at the end of the hospital stay.

**Table 2:** Patient characteristics at teriparatide initiation and during follow-up.

**N°**	**Diagnosis**	**Age at diagnostic (years)**	**Age at rh-PTH intro (years)**	**Indication**	**Calcium element (g/day)**	**1-α-calcidol (μg/day)**	**Sevelamer carbonate (mg/day)**	**rh-PTH (1–34) dose (μg/kg/day)**					**1-α-calcidol reintro**	**NC at baseline**	**NC at LF**
								**Intro**	**6 months**	**12 months**	**LF**	**Max**			
(1)	APECED syndrome	3.5	4.1	Symptomatic hypocalcemia despite optimized SOC (malabsorption)	6.2	3	4,800	0.6	1.2	1.5	1.1	1.5	No	Yes, grade 1	No
(2)	APECED syndrome	6.5	6.9	Symptomatic hypocalcemia despite optimized SOC (malabsorption)	3.2	2	6,000	1.8	1.4	1.4	2.0	2.0	Yes	Yes, grade 1	Yes, grade 1
(3)	CaSR mutation	1.5	7.6	Non-compliance + muscular symptoms	1.2	0.5	800	1.5	1.4	1.3	1.0	1.5	No	Yes, grade 2	Yes, grade 2
(4)	CaSR mutation	8.5	9.4	Non-compliance + symptomatic hypocalcemia	0.8	1.2	0	1.2	0.6	1.2	1.3	1.3	Yes	NA	Yes, grade 2
(5)	CaSR mutation	2.7	10.1	Non-compliance	0.4	0.5	2,400	1.5	1.4	1.3	0.4	1.5	No	Yes, grade 1	Yes, grade 2
(6)	APECED syndrome	3.7	11.2	Symptomatic hypocalcemia despite optimized SOC (malabsorption), non-compliance	3.6	1.5	7,200	1.0	0.5	0.5	0.8	1.0	Yes	No	No
(7)	CaSR mutation	0.1	11.5	Non-compliance	0.4	0.25	2,400	1.4	1.4	1.3	0.5	1.4	No	Yes, grade 1	Yes, grade 1
(8)	Post-thyroidectomy	10.8	13.0	Non-compliance	2.9	2	7,200	0.6	0.5	0.8	0.2	0.8	Yes	No	No
(9)	Di George syndrome	12.3	15.5	Non-compliance	1.2	3	7,200	0.9	1.3	1.7	0.8	1.7	Yes	Yes, grade 1	Yes, grade 1
(10)	Unknown, negative extended genetic analysis	14.3	15.5	Non-compliance	1.6	1.7	7,200	0.6	0.3	0.6	0.6	0.6	No	NA	Yes, grade 1

*No, number; APECED, autoimmune polyendocrinopathy candidiasis ectodermal dystrophy; CaSR, Calcium sensing receptor, rh-PTH, teriparatide; Intro, Introduction, LF, last follow-up, NC, nephrocalcinosis; SOC, standard of care**;** rhPTH (1–34), teriparatide; NA, not available*.

The pen device was used when giving 20 μg of teriparatide, explaining the constant initiation dose. After that, we propose adjustments of doses with a protocol using insulin serynges and small volumes to ensure that patients receive the expected dose of teriparatide. To obtain 5, 10, or 15 μg doses of teriparatide, Forsteo^®^ 20 μg/80 μl pen was used and patients have to take teriparatide drug with a 30 IU insulin syringe. In our local protocol, we describe teriparatide equivalence doses to obtain prescribed daily dose (for example: graduation 2 IU of insulin syringe corresponds to 5 μg of Forsteo® Graduation 4 IU of insulin syringe corresponds to 10 μg of Forsteo®…).

### Biochemicals

As part of the routine follow-up of these patients with hypoparathyroidism, total calcium at H0 (i.e., just before injection) and H4 (i.e., 4 h after teriparatide injection), ionized calcium, phosphate, magnesium, alkaline phosphatase (total ALP), PTH, creatinine, 25-OH vitamin D (25-D), C-terminal Fibroblast Growth Factor 23 (FGF23), osteocalcin (OC), and C-terminal Telopeptide of type 1 collagen (CTX) levels were regularly assessed. Local normal reference values for these biomarkers and several kits used have been previously reported in VITADOS study (18). Due to the physiological evolution of plasma phosphate and FGF23 during childhood, their levels were normalized and expressed as *Z*-score for age (19, 20). Estimated Glomerular Filtration Rate (eGFR) was estimated using the 2009 Schwartz formula (21). Laboratory parameters were collected at six different time points: initiation of teriparatide therapy (baseline), and after 1, 3, 6, and 12 months; data at last follow-up were also recorded.

### Imaging

Dual X-ray absorptiometry (DXA) of the spine and total body was performed (Hologic discovery densitometer, QDR APEX V8.26a; HOLOGIC Inc). Bone mineral density (BMD) was evaluated with spine *Z*-score adjusted for age, height and weight at teriparatide initiation and after 12 months of treatment, as previously reported (22). Renal ultrasounds (when available, *N* = 8 at baseline and *N* = 10 at 1 year) were all reviewed by an experienced pediatric radiologist (SC).

### Ethics

The study was approved by the local ethical committee (*Comité d'Ethique des Hospices Civils de Lyon*, session 27/06/2019, approval 19–84), and declared to the Information Technology and Liberty Commission (n°19–168).

### Statistical Analysis

Non-parametric Kruskal–Wallis tests followed by Dunn's tests were used. In all cases, *p*-values below 0.05 were considered statistically significant using GraphPad Prism software 8.0 (GraphPad, La Jolla, CA, USA). Results are presented as median (inter-quartile range, i.e., 25th−75th percentile) for biochemical routine parameters.

## Results

### Teriparatide Introduction

Relevant demographic, clinical and biomarkers features of the 10 patients (4 girls) at the time of teriparatide initiation are presented in Table 2. At a median age of 10.7 (8.1–12.6) years, eGFR was 110 (95–118) mL/min/1.73 m^2^, calcium level was 1.87 (1.81–1.96) mmol/L, phosphate level was 2.35 (2.17–2.50)mmol/L corresponding to age-standardized phosphate of 3.8 (2.5–4.9) SDS and 25-D levels were 77 (69–98) nmol/L. Only eight children had imaging at baseline. Nephrocalcinosis was found in 75% (*N* = 1 grade 1; *N* = 5 grade 2). Teriparatide therapy was introduced in all patients at the dose of 20 μg twice daily (12 h apart), corresponding to a median dose adjusted to body weight of 1.1 (0.7–1.5) μg/kg per day. For eight patients, teriparatide was introduced secondary to non-compliance of conventional treatment. All patients had at least a 1-year follow-up. The median time of follow-up was 2.8 (2.2–3.0) years.

### Evolution of Calcium, Phosphate, and 25-D Levels in Response to Teriparatide

**Figure 1:**
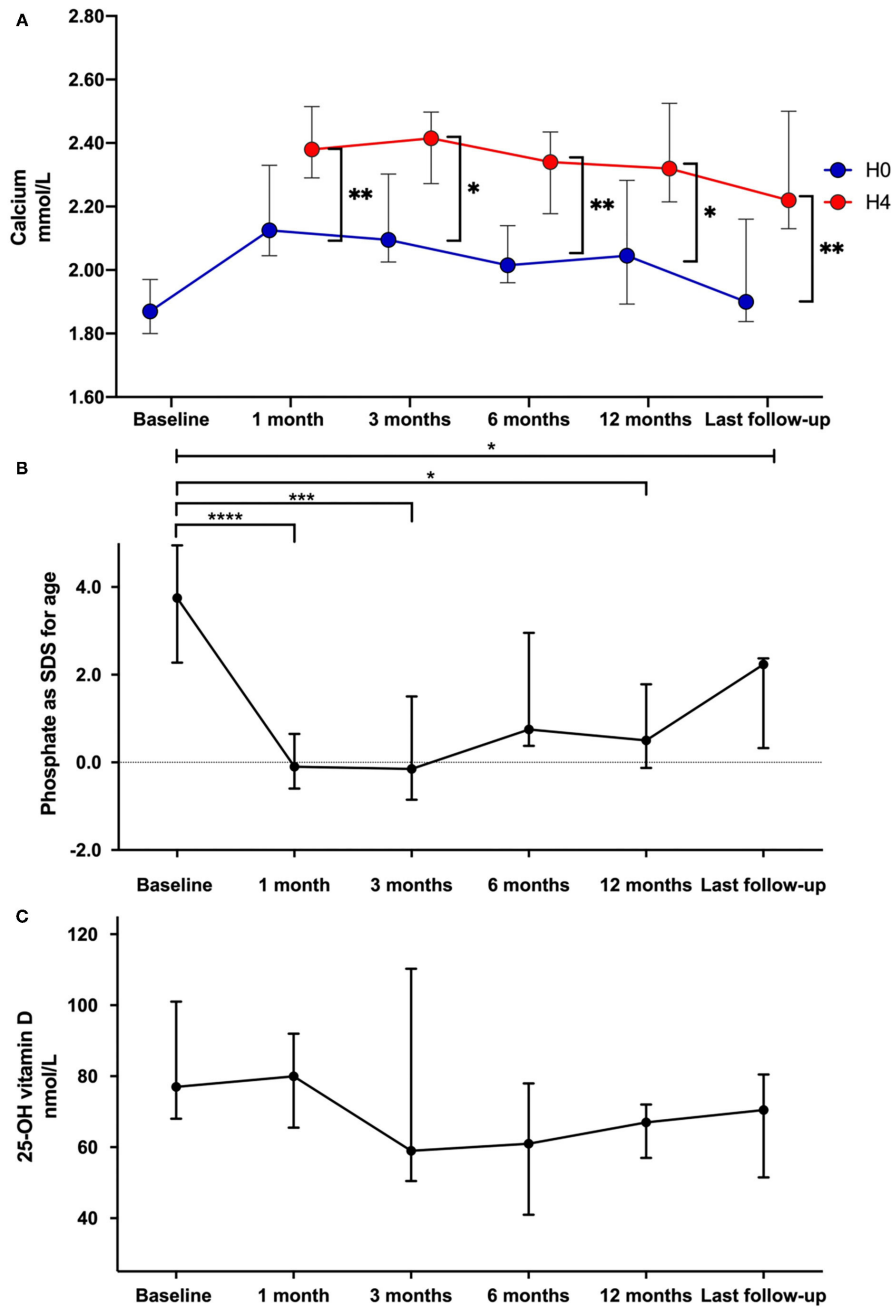
Comparison of total calcium **(A)**, phosphate levels as *z*-score for age **(B)**, and 25-OH vitamin D **(C)** data at teriparatide initiation and during follow-up. Each dot on the graph represents median with interquartile range of different biomarkers levels at the different time-points. Statistical analyses were performed with Kruskall Wallis test: *p < 0.05, **p < 0.01, ***p < 0.001, and ****p < 0.0001.

We observed a significant increase in calcium levels between H0 and H4 at all time points; calcium levels at H0 did not significantly increase as compared to baseline at all time points but as expected, calcium levels remained stable within the lower normal range or just below (Figure 1A). Of note, five children required the reintroduction of low doses of active vitamin D analogs during the follow-up in addition to native vitamin D supplementation. Indeed, low doses of active vitamin D allowed us to keep the same dose of teriparatide (so as to avoid pen deconditioning) in children displaying low calcium levels in the morning. As shown in Figure 1B, there was a significant decrease of phosphate levels from 3.8 (2.5–4.9) to 0.5 (−0.1 to 1.5) SDS after 12 months of teriparatide (*p* = 0.01). The evolution of the main biomarkers included phosphate levels are summarized in Table 3. FGF23 and FGF SDS remained stable under teriparatide treatment. As shown in Figure 1C, 25-D levels did not change throughout the observational period. As a result, during the first 12 months, patients received native vitamin D as a total of 240,000 (160,000–400,000) IU of cholecalciferol through intermittent supplementation (80–100,000 IU per dose). Reference values for phosphate, calcium and 25 OH vitamin D levels in children and adults were reported in Table 4.

**Table 3:** Summary of the biomarkers available in patients at the different follow-up.

	**Baseline**	**1 month**	**3 months**	**6 months**	**12 months**	**Last follow-up**
**Height SDS**	−0.1 (−1.1 to 1.6)	−0.1 (−1.1 to 1.4)	0.1 (0–0.5)	−1.0 (−2.0 to 0.1)	−2.0 (−2.0 to 0.1)	−0.1 (−1.1 to 1.1)
**Weight SDS**	0.1 (−1 to 0.5)	0.1 (−1.0 to 0.5)	−1.0 (−2 to 0.5)	0.1 (−1.0 to 0.5)	0.3 (−2.0 to 0.7)	0.3 (−1.0 to 0.5)
**Dose (μg/kg/j)**	1.1 (0.7 to 1.5)	1.1 (0.7 to 1.4)	0.8 (0.6 to 1.2)	1.3 (0.6 to 1.4)	1.3 (0.9 to 1.4)	0.8 (0.5 to 1.1)
**eGFR (mL/min** **/1.73 m**^2^**)**	110 (95 to 118)	140 (116 to 101)	124 (110 to 141)	121 (104 to 142)	108 (90 to 122)	107 (89 to 116)
**Calcium H0 (mmol/L)**	1.87 (1.81 to 1.96)	2.13 (2.06 to 2.30)	2.10 (2.04 to 2.23)	2.02 (1.97 to 2.11)	2.05 (1.91 to 2.22)	1.90 (1.84 to 2.13)
**Calcium H4 (mmol/L)**		2.38 (2.30 to 2.48)^**^	2.42 (2.32 to 2.49)^*^	2.34 (2.21 to 2.43)^**^	2.36 (2.23 to 2.48)^*^	2.22 (2.20 to 2.41)^**^
**PO4 (mmol/L)**	2.35 (2.17 to 2.50)	1.59 (1.44 to 1.65)	1.57 (1.49 to 1.86)	1.77 (1.68 to 2.00)	1.68 (1.53 to 1.90)	1.88 (1.77 to 2.08)
**PO4 (** ***Z*** **-score)**	3.8 (2.5 to 4.9)	−0.1 (−0.4 to 0.5)****	−0.2 (−0.6 to 1)^***^	2.0 (0.8 to 2.1)	0.5 (−0.1 to 1.5)^*^	2.2 (0.8 to 2.4)
**25-D (nmol/L)**	77 (69 to 98)	80 (66 to 89)	59 (54 to 72)	62 (47 to 73)	68 (63 to 74)	71 (56 to 103)
**FGF23 (RU/mL)**	171 (130 to 233)	391 (199 to 601)	102 (102 to 102)	112 (92 to 154)	108 (69 to 161)	148 (110 to 181)
**FGF23 (** ***Z*** **-score)**	2.3 (1.6 to 2.7)	4.3 (3.8 to 5.3)	1.1 (1.1 to 1.1)	2.1 (0.8 to 2.5)	1.1 (−0.2 to 2.5)	NA
**ALP (UI/L)**	223 (213 to 250)	287 (243 to 307)	275 (240 to 306)	285 (256 to 362)	294 (210 to 368)	304 (221 to 346)
**OC (μg/L)**	64 (51 to 70)	86 (72 to 90)	152 (152 to 152)	148 (125 to 240)^**^	134 (130 to 211)^**^	160 (128 to 211)^***^
**CTX (pg/mL)**	1,867 (1,763 to 2,264)	1,786 (1,283 to 2,440)	3,404 (2,588 to 4,221)	1,466 (1,335 to 1,726)	1,320 (959 to 1,893)	2,444 (1,583–3,110)
**U Ca/creat (mmol/mmol)**	0.5 (0.3 to 0.6)	0.9 (0.3 to 1.1)	0.7 (0.3 to 0.7)	0.5 (0.2 to 0.7)	0.7 (0.7 to 1.1)	0.6 (0.3 to 0.7)
**CaU (mmol/L)**	3.2 (2.1 to 6.8)	6.3 (2.6 to 10.1)	3.8 (2.8 to 4.3)	2.9 (1.7 to 4.6)	2.5 (2.3 to 4.6)	4.7 (1.8 to 6.4)
***Z*** **-score spine**	−0.6 (−1.0 to −0.1)	NA	NA	NA	1.3 (1.1 to 1.7)	1.0 (0.2 to 2.9)

*Intro, Introduction; eGFR, Estimated Glomerular Filtration Rate; PO4 Z-score, phosphate Z-score; 25-D, 25-OH vitamin D; CaU/creatU, urinary calcium/creatinine ratio; ALP, total Alkaline Phosphatase; OC, osteocalcin; CTX, C-terminal Telopeptide of type 1 collagen; NA, not available. Gray cells represent statistically significant difference of biomarkers between initiation and follow-up after commencing teriparatide. Results are presented as median (inter-quartile range, i.e., 25th−75th percentile) for biochemical routine parameters*.

* *p < 0.05*,

** *p < 0.01, and*

*** *p < 0.001*.

**Table 4:** Reference values for phosphate, calcium and 25 OH vitamin D levels in children and adults (11).

**Age range**	**Calcium (mmol/L)**	**Phosphate (mmol/L)**	**25-OH Vitamin D (mmol/L)**
Birth-5 months	2.18–2.83	1.50–2.40	75–120
6–12 months	2.18–2.75	1.50–2.40	75–120
1–5 years	2.35–2.70	1.50–2.10	75–120
6–12 years	2.35–2.58	1.20–1.90	75–120
13–20 years	2.20–2.55	0.70–1.50	75–120

### Evolution of eGFR and Urinary Calcium in Response to Teriparatide

**Figure 2:**
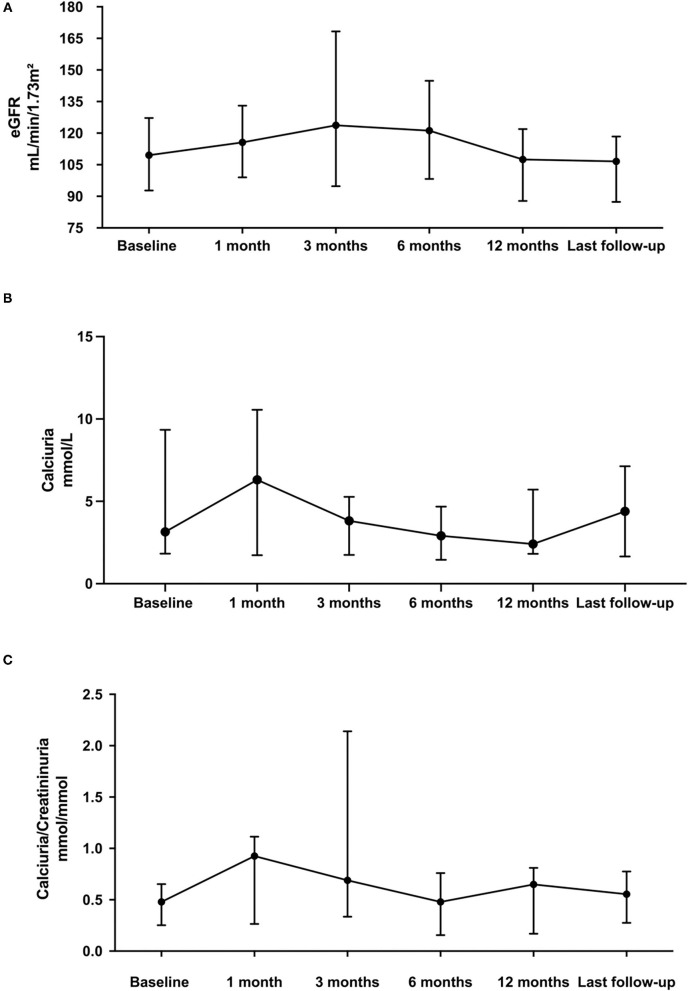
Comparison of eGFR **(A)**, calciuria **(B)**, and urinary calcium/creatinine ratio **(C)** on one voiding sample data at teriparatide initiation and during follow-up. Each dot on the graph represents median with interquartile range of biomarker levels at the different time-points. Statistical analyses were performed with Kruskall Wallis test: *p*, not statistically significant (NS).

Both eGFR, urinary calcium and urinary calcium/creatinine ratio remained stable with no statistically significant differences between the initiation of teriparatide and the last follow-up (Figures 2A–C).

### Bone Metabolism

**Figure 3:**
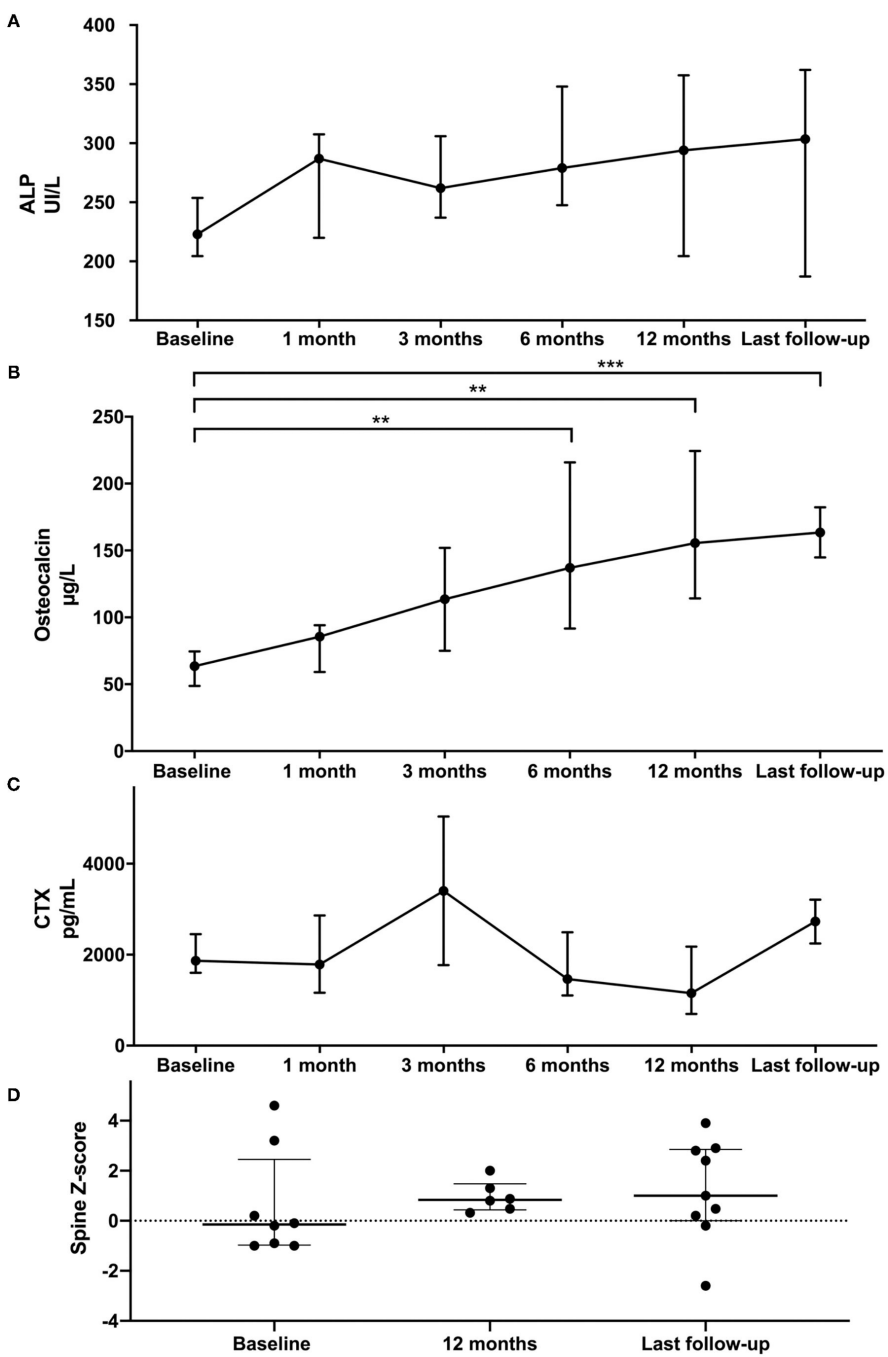
Comparison of ALP **(A)**, osteocalcin **(B)**, CTX **(C)**, and DXA spine **(D)** data at teriparatide initiation and during follow-up. Each dot on the graph represents median with interquartile range of biomarker levels at the different time-points. Statistical analyses were performed with Kruskall Wallis test: **p < 0.01 and ***p < 0.001.

As illustrated in Table 3 and Figure 3A, total ALP remained stable within the normal range during different follow-ups. We observed a significant increase of osteocalcin after 6 months with teriparatide treatment, as shown in Figure 3B. Regarding bone resorption, CTX remained stable during the entire follow-up (Figure 3C). Using DXA, spine *Z*-score remained stable during the follow-up (Figure 3D).

### Side Effects

Severe side effects were reported in one child, namely two episodes of severe symptomatic hypocalcemia and one of iatrogenic hypercalcemia; in this child, we had decided to begin teriparatide using a continuous sub-cutaneous pump, as previously described by others. However, because of the absence of clinical signs in the presence of severe hypocalcemia (as demonstrated by modifications on the electro-cardiogram) and the absence of alarms on the pump, we decided to switch the patient to intermittent twice-daily teriparatide after 4 months. These episodes of severe hypo- and hyper-calcemia occurred during the switch. One teenager also presented with ageusia with a complete neurological and Ear-Nose-Throat negative evaluation; symptoms completely disappeared with the decrease of teriparatide doses. One patient complained of muscular pain just after the injection, with a failure of switching back to conventional therapy (severe uncontrolled hypocalcemia). No bone symptoms were reported, excepted two teenagers with transient lumbar pain that resolved spontaneously.

### Renal Ultrasounds

On renal ultrasound, no nephrolithiasis was reported and two additional patients who had no imaging at baseline showed moderate nephrocalcinosis on US imaging at year 1. Among children with pre-existing nephrocalcinosis, one worsened from stage 1 to 2, while the other one remained stable or disappear for one patient.

## Discussion

This retrospective observational study showed intermittent bi-daily sub-cutaneous teriparatide therapy (1) allows a better control of calcium levels, without any changes in urinary calcium, (2) significant decreases phosphate levels that may have crucial long-term consequences for improving the cardiovascular risk of these children with a life-long chronic disease, and (3) is safe and effective with a better compliance (as compared to conventional therapy).

Pharmacokinetic analyses performed in osteoporotic women showed that daily subcutaneous injection of teriparatide 20 μg increased serum calcium levels with a maximal effect observed 4 h after the administration (23). In our study, we did not observe “hypercalcemia” at H4 but the increase between H0 and H4 was significant at all time points. Indeed, our management with teriparatide aims to obtain calcium levels in the “safety” area just below or within the lower normal range (“the lower the better”), and to decrease as much as possible fasting calcium levels without clinical symptoms of hypocalcemia, so as to avoid hypercalciuria and subsequent long-term side effects such as nephrocalcinosis.

We show that teriparatide maintained adequate calcium levels in the low-normal range or just below the normal range, with a significant increase 4 h after injection. However, five children required the reintroduction of low doses of vitamin D analogs during the evening to stabilize fasting calcium levels to maintain 25-D levels between 75 and 120 nmol/L, and supplementation was modified accordingly.

In pediatrics, data are scarce on the use of teriparatide, as summarized in Table 5 (6–15). Renal function remained stable, similarly to previous papers reporting no direct effects of teriparatide on neither renal function nor urinary calcium (6–8, 10, 11, 13–15). The effects of teriparatide on calciuria and subsequent nephrocalcinosis/lithiasis remain nevertheless likely on the long-term, at least theoretically, consistent with what is observed with conventional therapy. A long-term study treating 14 children with twice or thrice-daily subcutaneous teriparatide injections showed less hypercalciuria after teriparatide introduction. However, nephrocalcinosis worsened in 5 patients out of 14 subjects whilst renal function remained normal (14). Computed tomography and ultrasound were used to visualize renal calcifications which also added to the lack of precision in follow-up results. One of the patients in this cohort had a regression of nephrocalcinosis according to follow-up ultrasound results (14). This progression observed in these patients was probably caused both by the cumulative effects of intravenous calcium administration required in case of acute symptomatic hypocalcemia, the unphysiological nature of intermittent PTH replacement by multiple daily injections, the genetic background, notably in case of CaSR mutations with hypocalcemia associated with hypercalciuria and the progression of the disease. In 2015, Levy et al. (24) reported that children with hypoparathyroidism treated with calcitriol and calcium supplements were at risk for nephrocalcinosis (38%; mean follow-up 7.4 years). It is nevertheless difficult to know whether nephrocalcinosis would have also worsened with conventional therapy, even though this is very likely. Moreover, one should not forget that the evaluation of nephrocalcinosis on renal ultrasounds, although inexpensive and harmless for the patient, can be operator-dependent and can only be “semi-quantitative” at best. In our series, moderate nephrocalcinosis appeared in two patients (without pre-therapeutic renal ultrasound). Among children with pre-existing nephrocalcinosis, one worsened from stage 1 to 2, while the other one remained stable.

**Table 5:** Summary of the pediatric studies reporting the use of teriparatide in children.

	**References**	**N° of patients**	**Study design**	**Age at inclusion (years)**	**Follow-up**	**Teriparatide dose**	**Main conclusions: calcium/phosphate homeostasis**	**Main conclusions: calciuria/** **nephrocalcinosis**	**Main conclusions: bone turnover**	**Side effects**
(1)	Winer et al. (6)	14	Randomized crossover trial	4–17	28 weeks	Once-daily regimen 58 μg/d; vs. twice-daily regimen 25 μg/d	Twice-daily regiment: more effective to reduces the variation in Ca levels	Normalized mean 24-h urine calcium excretion		No severe hypocalcemia or hypercalcemia
							No significant differences in the serum Ph			
(2)	Winer et al. (7)	12	Randomized crossover trial	5–14	3 years	Twice-daily regimen 0.6 μg/kg/day vs. calcitriol	Serum calcium levels were maintained at, or just below, the normal range	Urine calcium levels maintained at, or just below, the normal range	Bone Mass Density did not differ	One episode of hypocalcemia
										Single report of bone pain
(3)	Linglart et al. (8)	3	Case reports	8; 11; 13	3 years	Continuous pump 2.6 μg/kg/d	Serum Ca: near-normal levels	urinary calcium: near-normal levels in three children	Bone Mass Density: normal values	Hypocalcemic seizure for one patient
(4)	Gafni et al. (9)	5	Randomized trial	15–49	18 months	Thrice-daily 0.06 μg/kg/d			hPTH 1–34 treatment increased cortical porosity; BMD *Z*-score was unchanged at the spine	
						Twice-daily 0.23 μg/kg/d				
(5)	Winer et al. (10)	12	Randomized controlled trial	7–20	13 weeks	Twice-daily 0.32 μg/kg/d vs. pump 0.11 μg/kg/d	Serum Ca: near-normal levels	Normalized mean 24-h urine calcium excretion	Reduction of markers of bone turnover with continuous pump	No severe hypocalcemia or hypercalcemia
(6)	Matarazzo et al. (11)	6	Self-controlled trial	6–18	2.5 years	Twice-daily 0.7 μg/kg/day	Mean blood calcium, phosphate, and alkaline phosphatase were not modified	Significant reduction of calciuria		Number of tetanic episodes reduced in four patients compared to conventional treatment
(7)	Gafni et al. (12)	9	Retrospective study	15–50	20–61 months	Thrice-daily 0.45 μg/kg/d			Bone-specific ALP, N-telopeptide, and osteocalcin increased significantly with hPTH 1–34; BMD was unchange	Four subjects discontinued hPTH 1–34 early; 1 because of bone pain; 3 because of a decline in radial BMD *T*-score or *Z*-score to < -2
						Twice-daily 0.37 μg/kg/d				
(8)	Saraff et al. (13)	4	Retrospective study	8–13	24 months	Continuous pump 0.4 μg/kg/d	Normalization and maintenance of serum calcium and phosphate	Normalization of urinary calcium	Increased ALP noted in the first 6 months on continuous pump, indicating an increase in bone turnover	No significant side effects
(9)	Winer et al. (14)	14	Observational study	7–16	6.9 years	Twice-daily 0.75 μg/kg/d		Nephrocalcinosis progressed in 5 of 12 subjects	Mean height velocity and lumbar spine, whole body, and femoral neck bone accretion velocities: normal ranges	One patient:tibial bone pain; four patients had headaches
(10)	Tuli et al. (15)	6	Self-controlled trial	15–20	9.2 years	Twice-daily 12.5 μg/d	Mean blood calcium, ALP, and phosphate did not significantly change	Significant reduction of the urinary calcium-to-creatinine ratio		No event

Phosphate is a vascular toxin and is often seen as a “silent killer” because of its dramatic effect on vascular calcifications and mortality, in general but also in CKD populations (23) and hypoparathyroidism patients (25). When vessels are exposed *in vitro* to high phosphate levels conditions, extensive calcification with hydroxyapatite deposition develop (26). In the present study, we demonstrated that phosphate levels significantly decreased with teriparatide therapy; as such, we hypothesize beneficial long-term effects on the development of ectopic calcifications in these patients (27). Hyperphosphatemia could be explained notably because of the phosphate overload in western diets that is mainly due to the phosphate “hidden” in food additives. When hyperphosphatemia is secondary to hypoparathyroidism, FGF23 levels usually increase to try to inhibit tubular phosphate reabsorption (28), as also illustrated there with increased FGF23 levels (that may also have been increased by active vitamin D analogs). High FGF23 levels are associated *per se* with left ventricular hypertrophy and increased morbi-mortality, because of a direct toxic effect of FGF23 on cardiomyocytes (28). Here, FGF23 levels did not change overtime, however FGF23 SDS was increased at baseline and we observed a (expected) trend toward a reduction in FGF23 levels under teriparatide.

In 2015, Gafni et al. (12) evaluated the effects of recombinant PTH on bone markers in nine teenagers and adults with hypoparathyroidism. Here, we report similar results, namely increased osteocalcin levels, with no modification of BMD. In growing rat models, rhPTH can induce bone tumors, such as osteoma, osteoblastoma, and osteosarcoma in a dose-dependent manner when high doses were used (29). Important to note, these doses were 3–71-time higher than the doses used in humans, and the exposition to the product was far longer. As such, it is difficult to transfer these data from rats to humans not only because of the differences in doses but also because of the differences in skeletal physiology between the species (29). We nevertheless believe that parents and caregivers must be aware of the “off-label” use of this drug in pediatrics, with a close follow-up and a special warning in case of bone pain.

Two recombinant human PTH are available, i.e., the 1–84 approved in the USA in 2015, and the 1–34 teriparatide evaluated here (30). These two compounds have a reported acceptable tolerability. Ageusia and oral hypoesthesia have been reported after rPTH 1-84 (31), but never to date with teriparatide. However, one teenager displayed ageusia that disappeared when teriparatide doses were decreased.

Despite the fact that we were unable to scientifically assess compliance and quality of life (QOL) of these children in this retrospective series of cases, seven were non-compliant at teriparatide initiation, and after teriparatide initiation, only one teenager still displayed a poor compliance, because of a very poor socio-familial support. Moreover, the majority of patients reported an improved quality of life with teriparatide therapy, explaining that fatigue significantly decreased with the disappearance of muscular symptoms. Of note, when we told the teenager with ageusia that it could be treatment-related, he did not accept to withdraw teriparatide, only to decrease the doses. Unfortunately, we unfortunately did not assess compliance and QOL using dedicated scales, and these points obviously deserve other studies.

Only two studies reported the use of teriparatide with a continuous sub-cutaneous pump (10, 13), as opposed to what is presented here. Although bi-daily sub-cutaneous administration of teriparatide represents a safe and effective long-term therapy for children, it has been discussed and hypothesized that the use of a continuous pump could be better, and more physiological, as illustrated in Table 5 (8, 10, 13). In 2014, Winer et al. have compared bi-daily administration to continuous pump delivery in a prospective cross-over trial. They concluded that the continuous administration was more physiological both from a calcium homeostasis and bone turnover point of view, with lower daily doses of teriparatide (10). Seven basal rates and seven bolus options were programmed to mimic homeostatic circulating PTH. However, for reasons of technical feasibility, mimicking circadian PTH secretion failed and hyperphosphatemia was observed during the continuous pump administration, as opposed to the bi-daily sub-cutaneous administration. In animal models, intermittent and continuous PTH increase bone formation independently of effects on bone resorption, but only intermittent PTH increases bone mass consistently (32). In children, osteocalcin, alkaline phosphatase and NTX-telopeptide decreased significantly after teriparatide pump treatment (10).

This study has some limitations, and notably the small number of patients. However, we are in the field of orphan diseases in pediatrics, with an off-label use of novel therapies.

In conclusion, our observational data suggest that teriparatide in pediatric hypoparathyroidism can be a useful tool to correct calcium but also phosphate levels, without major side effects. Even though national guidelines already propose this management as a second-line off-label therapy in these children, notably in France, larger international studies may evaluate its long-term consequences especially for the potential risk of hypercalciuria and nephrocalcinosis later in life, and its interest to improve the quality of life of these children with life-long chronic diseases.

## Data Availability Statement

The raw data supporting the conclusions of this article will be made available by the authors, without undue reservation.

## Ethics Statement

The studies involving human participants were reviewed and approved by Local Ethical Committee (Comité d'Ethique des Hospices Civils de Lyon, session 27/06/2019, approval 19-84), and declared to the Information Technology and Liberty Commission (n°19-168). Written informed consent to participate in this study was provided by the participants' legal guardian/next of kin.

## Author Contributions

JBe and SF have written the manuscript. SC, SL, RC, AM, and AB-T have read and corrected the manuscript. All authors contributed to the article and approved the submitted version.

## Funding

JBe received an educational grant from the ORKiD (Orphan Kidney Diseases *Filière Maladies Rares*) Network.

## Conflict of Interest

The authors declare that the research was conducted in the absence of any commercial or financial relationships that could be construed as a potential conflict of interest.

## Publisher's Note

All claims expressed in this article are solely those of the authors and do not necessarily represent those of their affiliated organizations, or those of the publisher, the editors and the reviewers. Any product that may be evaluated in this article, or claim that may be made by its manufacturer, is not guaranteed or endorsed by the publisher.
